# Effects and Mechanisms of Surface Topography on the Antiwear Properties of Molluscan Shells (*Scapharca subcrenata*) Using the Fluid-Solid Interaction Method

**DOI:** 10.1155/2014/185370

**Published:** 2014-05-28

**Authors:** Limei Tian, Ximei Tian, Guoliang Hu, Yinci Wang, Luquan Ren

**Affiliations:** ^1^Key Laboratory of Bionic Engineering (Jilin University), Ministry of Education, Changchun 130022, China; ^2^Secretariat of the International Society of Bionic Engineering, Jilin University, Changchun 130012, China; ^3^School of Automobile and Traffic Engineering, Jiangsu University, Zhenjiang 212013, China

## Abstract

The surface topography (surface morphology and structure) of the left *Scapharca subcrenata* shell differs from that of its right shell. This phenomenon is closely related to antiwear capabilities. The objective of this study is to investigate the effects and mechanisms of surface topography on the antiwear properties of *Scapharca subcrenata* shells. Two models are constructed—a rib morphology model (RMM) and a coupled structure model (CSM)—to mimic the topographies of the right and left shells. The antiwear performance and mechanisms of the two models are studied using the fluid-solid interaction (FSI) method. The simulation results show that the antiwear capabilities of the CSM are superior to those of the RMM. The CSM is also more conducive to decreasing the impact velocity and energy of abrasive particles, reducing the probability of microcrack generation, extension, and desquamation. It can be deduced that in the real-world environment, *Scapharca subcrenata's* left shell sustains more friction than its right shell. Thus, the coupled structure of the left shell is the result of extensive evolution.

## 1. Introduction

**Figure 1:**
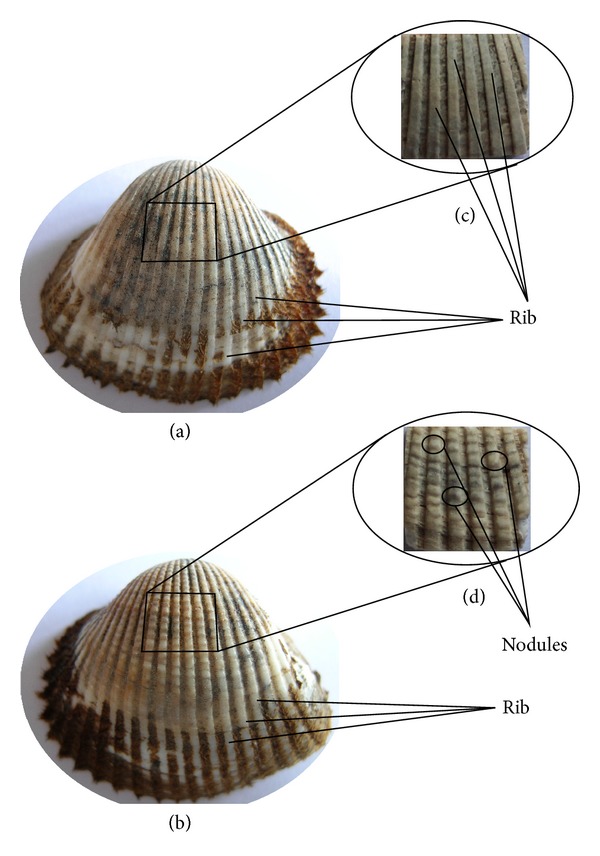
The morphology and structure of* Scapharca subcrenata*: (a) right shell; (b) left shell; (c) experimental sample illustrating rib morphology of right shell; and (d) experimental sample illustrating coupled structure of left shell.

In one hundred million years of evolution, biological materials and formations have often developed optimised properties and organisations [1–4], forming material topological structures that have allowed adaptation to various stress states [5]. These structures usually accord with the principle of maximum efficiency, using the least amount of material and the simplest structures to improve wear resistance through surface texture optimisation. Intertidal shellfish are one of the most typical examples. Made of a natural mineralisation material, the structures of their shells are assembled in ways that reflect the best fit between strength and toughness [2, 3]. Shells have complex surface morphologies and structures that commonly exhibit good wear resistance properties [6]. The dry sliding wear resistance of some shells can even be compared with that of diamond-like carbon coatings [7]. Research on shellfish has mainly focused on microstructural characteristics and mechanical properties [8–13], chemical composition, and the mechanical properties of shells' pearl layer and its mineralisation mechanism [14–17]. Some progress in wear mechanism research has been made through focusing on wear mechanisms under different external factors or preliminary work on the corresponding wear-resisting performance of bionic samples [5, 18–22]. In fact, the morphology and structures of some molluscan shell surfaces differ from the left to the right shell—a phenomenon that influences the wear-resistant properties but has failed to receive enough attention from the materials development community. For example, the typical shellfish* Arca subcrenata* (*Scapharca subcrenata*), which belongs to the phylum Mollusca, class Bivalvia, suborder Taxodonta, and family Arcidae, has a shallow, buried habitat, living in the sandy mud of shallow seas within a depth of 20 metres. During its lifecycle,* Scapharca subcrenata* is exposed to a certain degree of abrasive wear and the surface morphology and structure of its left shell are different from those of its right shell (Figures 1(a)-1(b)). The right shell is smaller and has an obvious radiation rib, giving it a rib morphology. The left shell is larger and has a rib with nodules that form a special coupled structure. Does this coupled structure influence antiwear ability? If so, what is the antiwear mechanism between the shells and the abrasive system? The objective of this study was to investigate how the rib morphology and coupled structure of the right and left shell surfaces of* Scapharca subcrenata* affect antiwear ability and to understand the mechanism between the morphology/coupled structure and the abrasive system. A contrast experiment was used to study the antiwear characteristics of the morphology and the coupled structure and the antiwear mechanism between them, by adopting a fluid-solid interaction (FSI) simulation method.

## 2. Experiment and Simulation Conditions

As Figures 1(a)-1(b) show,* Scapharca subcrenata's *right shell has a different morphology and structure than those of its left shell. The right shell has a rib morphology while the left has a coupled structure (formed by ribs and nodules through a specific coupling mechanism), and this very likely influences their overall antiwear performance. Thus, a simple contrast experiment should clarify their respective antiwear characteristics.

### 2.1. Antiwear Ability of Topography: A Simple Contrast Experiment

#### 2.1.1. Test Description

**Figure 2:**
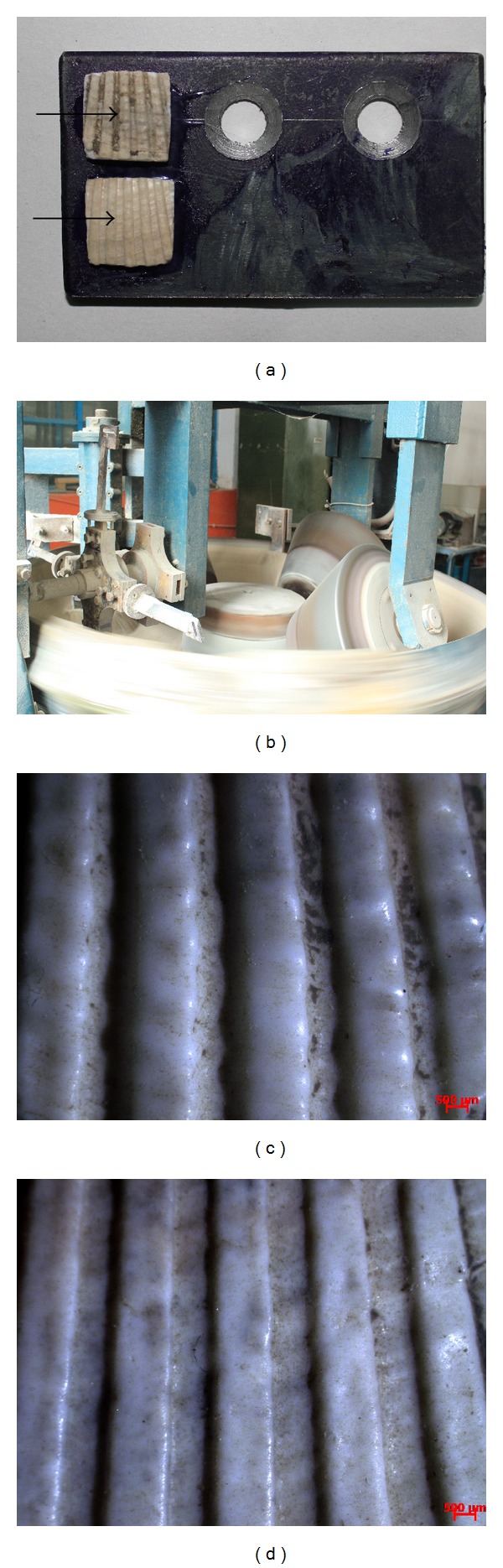
Illustration of the antiwear test equipment and results of two samples: (a) shell examples pasted on the clamp; the arrow indicates the abrasive direction; (b) JMM rotating disc soil abrasive wear testing machine; (c) stereoscopic microscope photo of worn coupled structure; and (d) rib morphology.

*Scapharca subcrenata* samples were taken from the mud beach of Tianjin, Tanggu, the soft parts were removed, and the shells were rinsed with clean water and dried naturally. A 10 mm × 10 mm example of each shell was cut from the area with the most uniform thickness and curvature (Figures 1(c)-1(d)) and then pasted to the clamp surface (Figure 2(a)) using a high-strength hardened gel. Measures were taken to ensure that each example's surface stood at about the same height off the clamp, and then the clamp was fixed in the bracket of a JMM rotating disc soil abrasive wear testing machine (Figure 2(b)).

In line with the actual living conditions of* Scapharca subcrenata*, the test parameters were as follows: abrasive adopted was quartz sand SiO_2_, sized from 0.42 to 0.84 mm; sliding velocity was 1.68 m/s; moisture content of abrasive was 3 wt%; attack angle of clamp was 24°; samples embedment depth was about 40 mm; and test environment temperature ranged from 18 to 22°C. The weight loss ratio of the samples was chosen as the experimental index. Sample quality was measured before and after wear using BSA224S-CW electronic scales with an accuracy of 0.01 mg. The weight loss ratio *η* can be calculated as

(1) $$\begin{array}{c} \eta =\frac{W_{a}-W_{b}}{W_{a}}\times 100\%, \end{array}$$

where *W*_*a*_ and *W*_*b*_ are the sample quality before and after wear, respectively, and *g* is the unit.

#### 2.1.2. Experimental Results and Analysis

The weight loss ratio of the rib morphology and coupled structure samples was calculated according to (1) and shown in Table 1.

As the stereoscopic microscope photos of the worn shell surfaces (Figures 2(c)-2(d)) show, there are no macro scratches or furrows even after a long time at a high strength of abrasive wear. Compared with the rib morphology example, the coupled structure example with ribs and nodules is well preserved, suggesting high abrasive wear resistance properties. However, Table 1 reveals that the average weight loss ratio for the coupled structure was 1.62% while that for the rib morphology was 2.30%, indicating that the antiwear abilities of the former were better than those of the latter. An *F* test showed that the wear weight loss ratio was significantly different between the morphology and coupled structure. Therefore, the antiwear capabilities of the* Scapharca subcrenata's* left shell were different from those of its right shell under the same conditions.

The remarkable antiwear performance of these topographies and their mechanisms were investigated by adopting a solid coupling simulation method.

### 2.2. Simulation 

#### 2.2.1. Established Simulation Model

**Figure 3:**
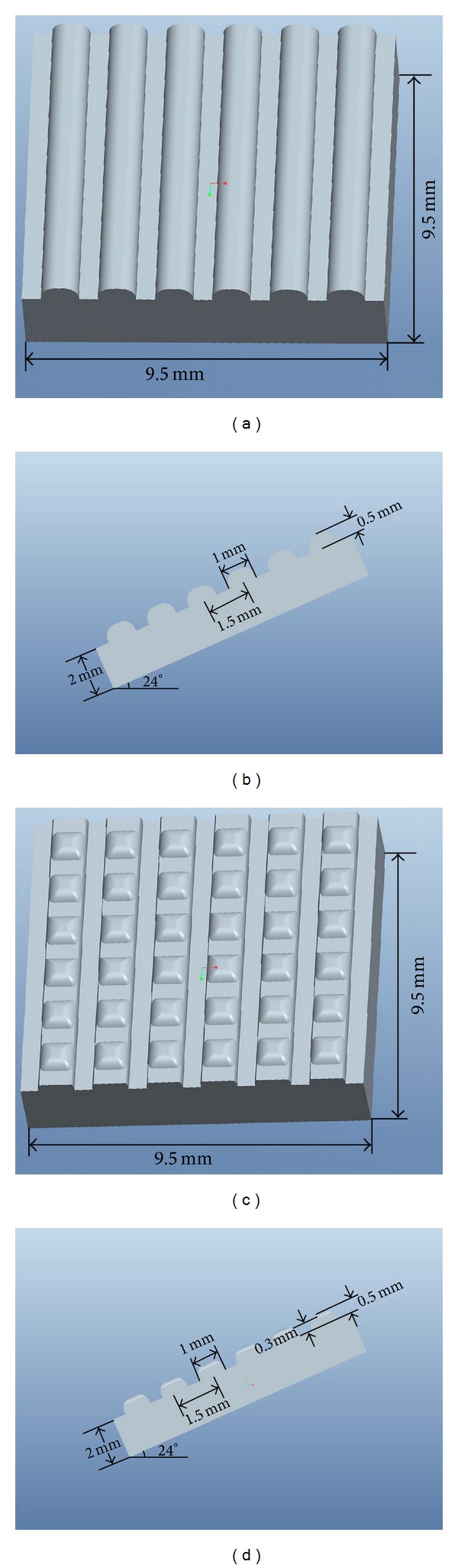
RMM and CSM and their parameters: (a) RMM mimics the right shell; (b) parameters of RMM, front view; (c) CSM mimics the left shell; and (d) parameters of CSM, front view.

Given the different surface topographies of the right and left shell examples (Figures 1(c)-1(d)) and without considering the microscopic structure of a cross-section and the composite of material, two simulation models were established using Pro/E software: the rib morphology model (RMM) and the coupled structure model (CSM). The rib formation and coupled structure were uniformly distributed on the simulation model, and the parameters for the RMM and CSM are shown in Figures 3(a)-3(d).

#### 2.2.2. Determination of Fluid Domain

**Figure 4:**
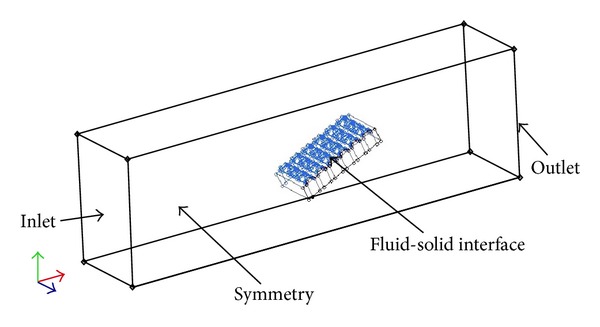
The computed field applied in the numerical simulation.

Based on the numerical simulation, the computation domain was set as cuboids (Figure 4). The length, width, and height of the domain were 5 times the length, 4 times the width, and 6 times the height of the model, respectively. The distance between the inlet and the front of the model was set as 2 times the length of the model. Regarding the model's symmetry, half was adopted for numerical simulation and analysis. Consistent with the experiment, the attack angle of the flow was set at 24° and the flow was perpendicular to the ribs.

#### 2.2.3. Discretisation of the Computation Domain

**Figure 5:**
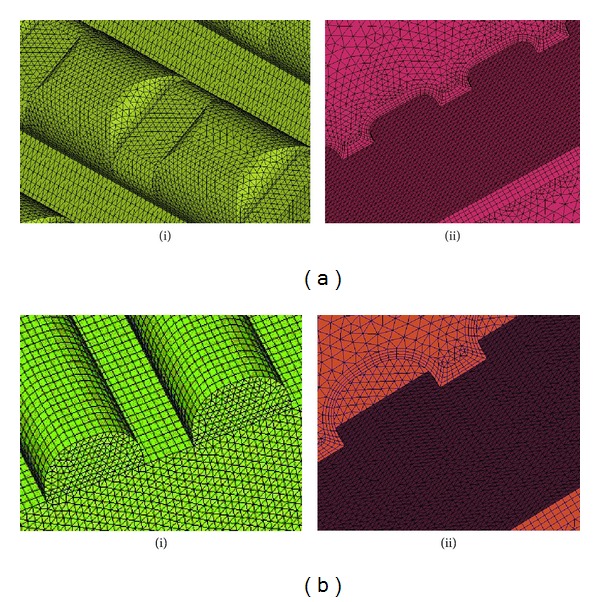
Local and interface meshes for solid and fluid domains: (a) CSM mesh, (i) local mesh of solid domain and (ii) interface mesh of fluid domain; (b) RMM mesh, (i) local mesh of solid domain and (ii) interface mesh of fluid domain.

The mesh model was processed using Hypermesh software. A tetrahedral mesh was adopted to represent the complex surface of the CSM (Figure 5(a)) while the RMM used a hexahedral mesh (Figure 5(b)). To improve the computational accuracy, four layers of boundary mesh were set at the interface of the fluid domain.

#### 2.2.4. One-Way Coupled Computational Platform

**Figure 6:**
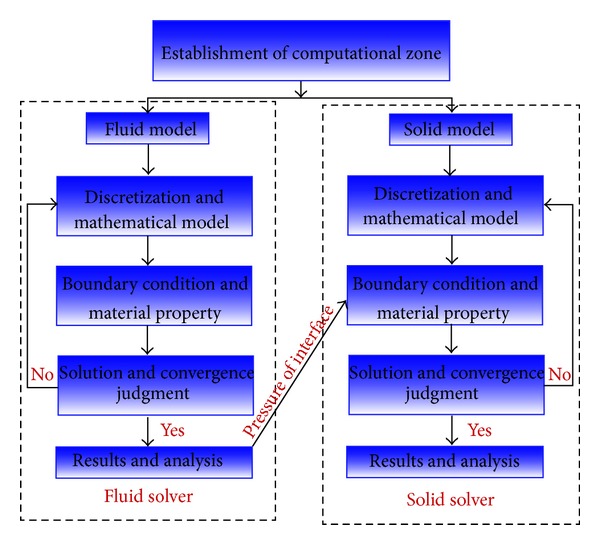
Calculation principle process based on Workbench.

A one-way coupled computational platform was established using the ANSYS Workbench software. The calculation principle is shown in Figure 6. First, the model was meshed using Hypermesh software. Second, the high-quality mesh was transported to CFX through the Finite Element Modeller module for steady calculation. Finally, the pressure results obtained at the interface of the fluid domain were imported into the static structure module as a boundary condition. Static structural calculation was performed with other boundary conditions to analyse the wear resistance characteristics of each model.

#### 2.2.5. Turbulent Model, Material Parameters, and Boundary Conditions

The fluid consisted of water and spherical quartz sand, with the latter accounting for 97% of the volume fraction. Water was set as the continual phase and quartz sand as the dispersed solid particles. The specific material parameters of the fluid can be seen in Table 2.

A two-phase homogenous *k* − *ω* turbulent model was adopted and two two-fluid models—including the mass and momentum conservation equations—were used to simulate the abrasive behaviour. The mass conservation equation can be expressed as

(2) $$\begin{array}{c} \frac{\partial }{\partial t}\left(r_{\alpha }\rho_{\alpha }\right)+\nabla \cdot \left(r_{\alpha }\rho_{\alpha }U_{\alpha }\right)=0, \end{array}$$

where *α* = *L*, *S* refer to the liquid and solid phases, respectively; ∇ is Laplace operator; *ρ* is fluid density; kg · m^−3^, *r* presents the volume fraction of fluid, %; and *U*_*α*_ refers to fluid velocity, m/s.

The momentum conservation equation is expressed as

(3) $$\begin{array}{c} \frac{\partial }{\partial t}\left(\rho U\right)+\nabla \cdot \left(\rho U⊗U-\mu \left(\nabla U+{\left(\nabla U\right)}^{T}\right)\right)=S_{M}-\nabla p, \end{array}$$

where fluid density can be expressed as *ρ* = *r*_*L*_ *ρ*_*L*_ + *r*_*S*_ *ρ*_*S*_, viscosity is *μ* = *r*_*L*_ *μ*_*L*_ + *r*_*S*_ *μ*_*S*_, *S*_*M*_ is the momentum source item caused by the external force, and ∇*p* refers to the turbulent pressure gradient.

The momentum transfer between the two phase relays on the interphase forces includes the impact of drag and lift. The Gidaspow (4) and Saffman (5) submodels are also represented in this study as

(4) $$\begin{array}{c} C_{D}=150\frac{{\left(1-\alpha_{l}\right)}^{2}\mu_{l}}{\alpha_{l}d_{s}^{2}}+\frac{4}{7}\frac{\left(1-\alpha_{l}\right)\rho_{l}\left|v_{l}-v_{s}\right|}{d_{s}},\;\alpha_{l}<0.8, \end{array}$$

where *C*_*D*_ is the liquid-solid drag coefficient and *α*_*l*_, *μ*_*l*_, and *ρ*_*l*_ are liquid phase volume fraction, dynamic viscosity, and density, respectively. *d*_*s*_ is the solid phase particle size and |*v*_*l*_ − *v*_*s*_| refers to the velocity gradient:

(5) $$\begin{array}{c} F_{s}=3.0844m_{s}\frac{{\left(\rho_{l}\mu_{l}\right)}^{0.5}}{\rho_{s}d_{s}}{\left|\nabla \left|v_{l}\right|\right|}^{0.5}\left|v_{l}-v_{s}\right|, \end{array}$$

where *F*_*s*_ is Saffman's lift, *m*_*s*_ is the solid particle quality, and *ρ*_*s*_ refers to solid particle density.

The inlet was set as the velocity boundary, the velocity and turbulence intensities being 1.68 m/s and 0.5%, respectively. It was also assumed that the water and quartz sand were well mixed at the inlet, without considering any external disturbances. The outlet was set as a constant pressure boundary, with a static pressure of 0 Pa. The model's middle surface was set as symmetry. The nonslip, smooth wall was applied at the interface and other surfaces. Based on the platform, the pressure results at the interface of the fluid domain were transported to the solid domain for structural strength analysis.

The material properties of the solid domain are shown in Table 3.

The bottom of the solid model was set as a fixed wall, the middle surface as symmetry, and the displacement as zero. The interface between the solid and fluid domains was fluid-solid and used to receive the pressure results at the interface of the fluid domain after the completion of static fluid calculation.

## 3. Results and Discussion

### 3.1. Fluid Domain

#### 3.1.1. Fluid Velocities of the Two Models and Their Antiwear Properties

**Figure 7:**
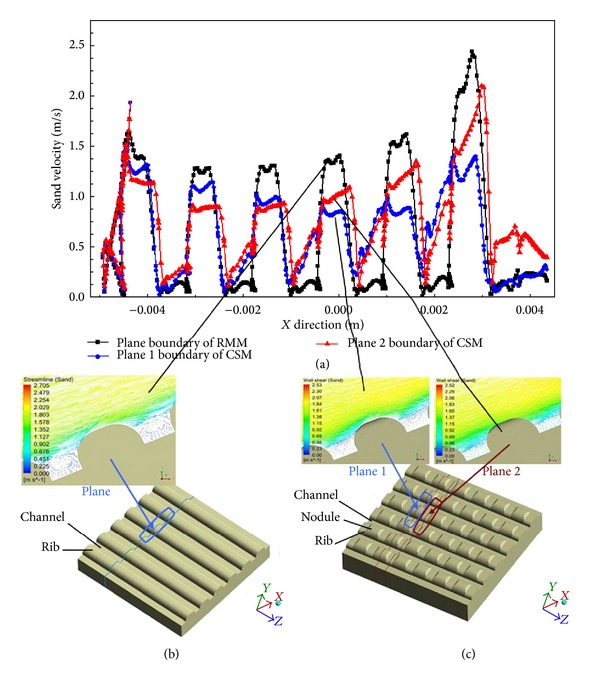
Fluid velocity of two models in *x* direction: (a) sand velocity on different plane boundaries of two models; (b) velocity vector on the plane of RMM; and (c) velocity vector on the plane 1 and plane 2 of CSM.

The relationship between the flow velocity and erosion rate can be expressed as

(6) $$\begin{array}{c} \varepsilon =kv^{n}, \end{array}$$

where *v* is flow velocity and *k* and *n* are constant, respectively. This kind of exponential relationship between erosion rate and flow velocity is unrelated to particles, material type, and the attacking angle. Thus, the flow velocity of the medium is the main aspect contributing to its cutting and erosion behaviour and plays a significant role in the erosion process.  Figure 7(a) is a variation diagram of the sand velocity at the different plane boundaries of each model. Figures 7(b) and 7(c) are the velocity vector diagrams of the two models on different planes. The three velocities exhibit similar trends in the flow direction (Figure 7(a)). The flow velocity increased dramatically at top of the ribs' morphology or the small nodules and dropped significantly at the bottom of the ribs. However, compared with the RMM, the CSM velocity obviously declined at the top of the ribs or the nodules and rose slightly at the bottom because the nodules further affected the velocity distribution of the sand.  Figure 7(b) shows the velocity vector on the plane for the RMM and the effects of the ribs, with vortices forming downstream in the channels between the ribs that accelerated the rolling motion of the sand. However, for the CSM, the nodules on the ribs interrupted the continuity of the flow fields (plane 2 in Figure 7(c)). The vortices generated by the nodules and ribs were not as strong as those observed in the RMM, so the sand velocity was significantly reduced in the CSM. From (2), it can be inferred that the erosion rate of the CSM was lower than that of the RMM. From this perspective, the CSM could improve the velocity gradient distribution on the model surface and then decrease the impact velocity or energy of the sand particles, avoiding microcracks in the subsurface even deeper than the surface of the model. Therefore, the effect of fluid velocity on the two models reveals that the CSM had better wear resistance characteristics than the RMM.

#### 3.1.2. Fluid Mechanics Parameters and Their Effects on Antiwear Properties

**Figure 8:**
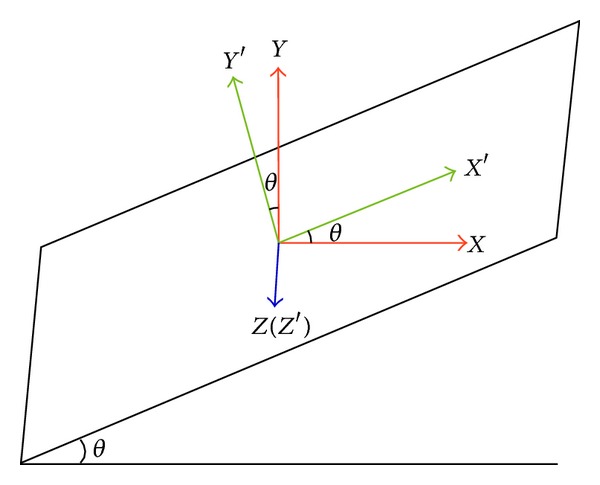
Schematic drawing showing coordinate transformation system.

*Fluid Mechanics Parameters of the Two Models.* Wear is a complex and dynamic process that includes abrasion, erosion, and minor cutting wear. Abrasive wear accounts for above 50% of overall wear. According to the Rabinowitsch model, *W* = *k*(*P*/*H*), where *W* refers to the wear rate and *P* and *H* refer to load and hardness of materials; the wear volume is proportional to the load on the surface and inversely proportional to material hardness within the unit slide distance. Thus, during the sliding process, the force of fluid directly determines the wear condition. Due to an attack angle of *θ*, the force of flow direction *F*_*X*_,  *F*_*Y*_, and *F*_*Z*_ should be transformed into *F*_*X*_′,  *F*_*Y*_′, and *F*_*Z*_′ through coordinate transformation (Figure 8), where *X*, *Y*, and *Z* represent the original coordinate system and *X*′, *Y*′, and *Z*′ represent the transformed coordinate system, with *θ* representing the attacking angle, which is set at 24°.

*F*_X_, *F*_Y_, and *F*_Z_ are, respectively, the component forces along the *X*-, *Y*-, and *Z*-axes directions on the original coordinate system, and *F*_*X*_′, *F*_*Y*_′, and *F*_*Z*_′ are the same forces after coordinate transformation. The conversion relationship is shown in (7). *F*_cutting  force  _ (8) and *F*_impact  force_ (9) are the cutting force and impact forces generated on the models' surfaces by the fluid and *F*_total  force_ (10) is the total force (cutting and impact forces, combined):

(7) $$\begin{array}{c} \left(\begin{array}{c} F_{X}^{\prime }\\ F_{Y}^{\prime }\\ F_{Z}^{\prime } \end{array}\right)=\left(\begin{array}{ccc} \cos\theta & \sin\theta & 0\\ \sin\theta & \cos\theta & 0\\ 0 & 0 & 1 \end{array}\right)\left(\begin{array}{c} F_{X}\\ F_{Y}\\ F_{Z} \end{array}\right), \end{array}$$

(8) $$\begin{array}{c} F_{\text{cutting}\;\text{force}}=\sqrt{{\left(F_{X}^{\prime }\right)}^{2}+{\left(F_{Z}^{\prime }\right)}^{2}}, \end{array}$$

(9) $$\begin{array}{c} F_{\text{impact}\;\text{force}}=F_{Y}^{\prime }, \end{array}$$

(10) $$\begin{array}{c} F_{\text{total}\;\text{force}}=\sqrt{F_{\text{cutting}\;\text{force}}^{2}+F_{\text{impact}\;\text{force}}^{2}}. \end{array}$$

According to the above equations, the two models' fluid mechanics parameters are shown in Tables 4 and 5.

Tables 4 and 5 show that the pressure force was the main cause of wear and that the pressure force of the CSM was smaller than that of the RMM—suggesting that, under the same conditions, the CSM was more conducive to drag reduction and thus had more wear resistance advantages than the RMM.

**Figure 9:**
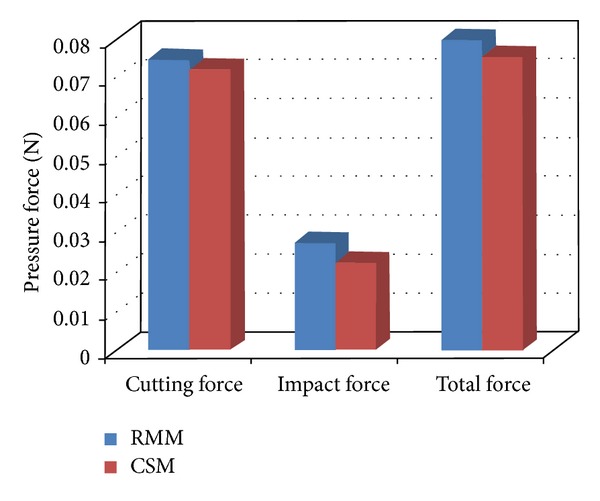
Histogram comparing the decomposed forces of pressure force acting on the models' surfaces.

*The Effect of the Models' Pressure Force on the Antiwear Properties.* Although the attack angle of the two models was the same, their pressure force differed (Tables 4 and 5), illustrating that it was not only related to the velocity and density of fluid, but also to the morphology and structure of the solid surfaces. The pressure force exerted on the models' surfaces was divided into tangential and normal forces, which represented the cutting and impact mechanisms of the wear, respectively. These two damage mechanisms had strong or weak interactions with each other depending on the attacking angle. When the attack angle was 24°, the horizontal components of the two models were stronger, making the cutting mechanism the main contribution to material loss. The force comparison figure for the two models (Figure 9) suggests that they were mainly bearing the cutting force in addition to a certain impact force. All three forces in the CSM were smaller than those in the RMM, indicating that the former had optimal wear resistance. If material effect is ignored as a factor, then the CSM should be adopted during the wear resistance design under this attacking angle.

#### 3.1.3. Shear Stress of the Two Models and Their Antiwear Properties

**Figure 10:**
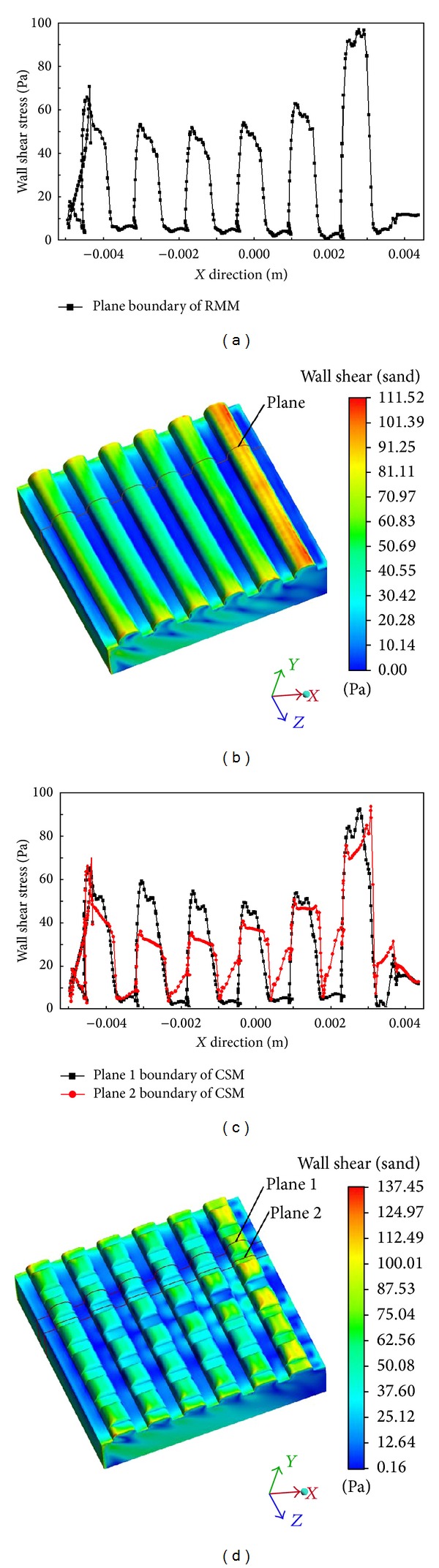
Shear stress nephogram and plane boundary curve of the two models: (a) shear stress curve in the plane boundary of the RMM; (b) shear stress nephogram of the RMM; (c) shear stress curve in the plane boundary of the CSM; and (d) shear stress nephogram of the CSM.

As the tangential force dominates the wear process, the shear stress at the interface is analysed here. The shear stress nephogram and comparison curves of the two models (Figure 10) revealed a similar trend in the shear stress on the interfaces of the two models. The values of shear stress on the ribs or nodules' surfaces largely exceeded the values of the shear stress between them. The sand particles directly contacted the windward sides of the ribs or nodules to produce a greater impact, dramatically increasing the shear stress curve. Although the particles' trajectories changed after crashing on the windward sides of the surfaces, the possibility that they would have direct contact with the leeward sides of the ribs or nodules decreased, sharply reducing the shear stress in those areas. Figures 7 and 10 infer that the nodules on the ribs acted like vortex generators, changing the trajectories of the particles and accelerating sand movement; that is, the sand was swept away quickly, which improved the shear stress distribution. Although the two models had the same surface material, the CSM had better antiwear performance than that of the RMM.

### 3.2. Contact Stress of Solid Domain

According to the strength theory of material mechanics, maximum contact stress prompts the degree of a model's yield failure. Therefore, the antiwear properties of the two models were analysed through the maximum tensile (first, main), maximum shear, and von Mises stresses. The peak stress represented the wear failure trend, so the antiwear properties of the two models were preliminary judged through the peak value of contact stress.  Table 6 presents the two models' peak stress values. All three peak stress values on the CSM surface were smaller than those for the RMM, suggesting that the CSM had better wear resistance properties than the RMM.

**Figure 11:**
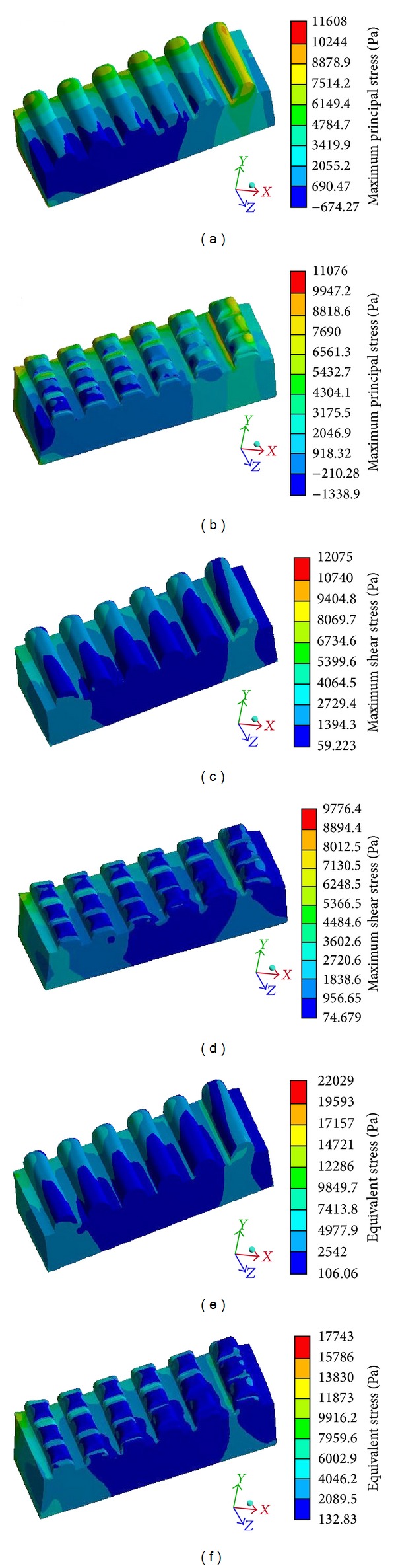
Different stress nephograms of the two models: (a) maximum principal stress of RMM; (b) maximum principal stress of CSM; (c) maximum shear stress nephogram of RMM; (d) maximum shear stress nephogram of CSM; (e) von Mises stress nephogram of the RMM; and (f) von Mises stress nephogram of the CSM.

The initiation of wear cracks is mainly caused by high tensile stress, and their extension is the result of the comprehensive effect of shear, tensile, and pressure stresses. The periodic ribs of the two models released stress periodically through the changes in abrasive particle trajectories. The three maximum stresses of the CSM (Figures 11(a), 11(c), and 11(e)) were more reasonable than those of the RMM (Figures 11(b), 11(d), and 11(f)). In particular, the von Mises stress (equivalent stress) in the CSM was significantly lower than it was in the RMM, indicating that the unit volume distortion energy density produced by the CSM was lower under the sand scouring condition. As a result, the possibility of crack occurrence and growth was effectively decreased, and the failure caused by the wear was also reduced.

These simulation results clearly suggest that the coupled structure effectively improved the antiwear performance of the shells tested, which is in accordance with the experimental results.

## 4. Mechanism Discussion and Summary 

**Figure 12:**
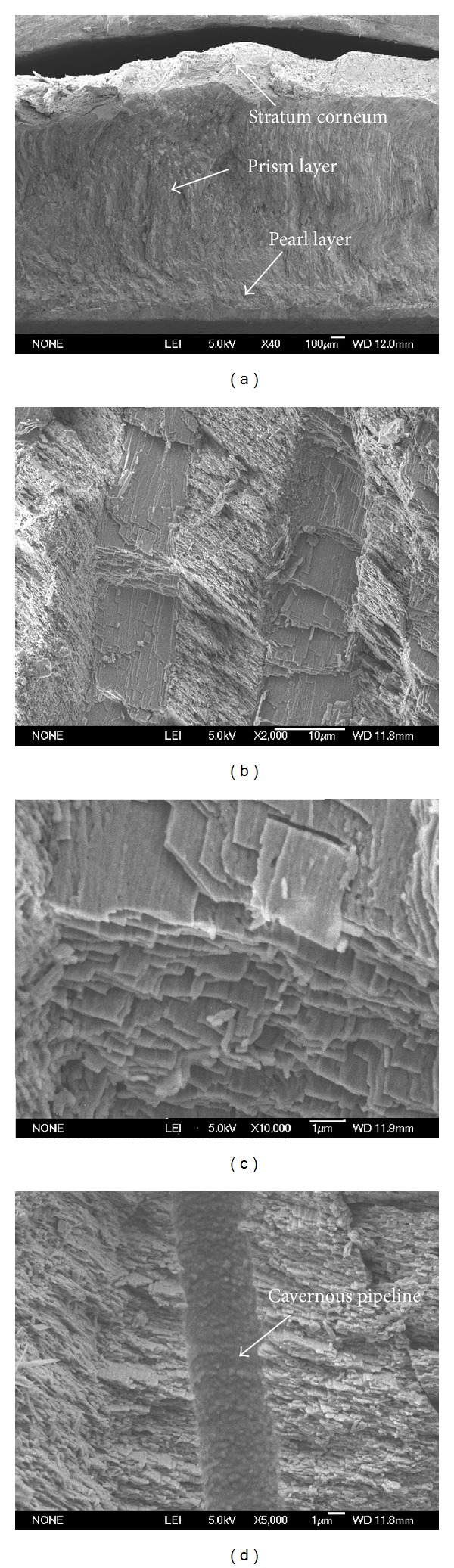
Microstructure of* Scapharca subcrenata* shell from cross-section: (a) the microstructure of the* Scapharca subcrenata* shell; (b) the first-order platelets of the crossed lamellar layer of prismatic layer; (c) the second-order lamellae of the crossed lamellar layer; and (d) the nacre layer and a typical pore canal tubule.

In fact, the wear resistance performance of molluscan shell also has important relationship with its microstructure. The cross-sectional microstructure of the* Scapharca subcrenata* shell is shown in Figure 12.

From the compared observation result of scanning electron microscopy (SEM), it can be found that the left and right shells of* Scapharca subcrenata *shell show the same microstructure, which is stratum corneum, prism layer, and pearl layer. The outer layer is relatively thick stratum corneum hard protein (shown in Figure 12(a)) and beneath is the prism layer occupying most of the shell. The prismatic layer takes on typical multilevel structure consisting of columnar structure on the whole, and adjacent columnar structure intersects vertically, presenting a typical crossed lamellar structure (see Figures 12(b)-12(c)). The organization of above complex structure has higher strength than a single layer structure, since this structure could absorb a lot of energy when impacted by external force, effectively restrains the expansion of the micro cracks, and avoids the appearance of fatigue wear and fracture micromechanism wear. The pearl layer is typical planar layered structure, distributed with a large number of cavernous pipelines (see Figure 12(d)) with a diameter of about 4 *μ*m, which may relate to the “stress slow-release effect,” and could restrain the crack initiation, thus playing an important role in antiwear performance. Obviously, the antiwear ability of* Scapharca subcrenata* shell has certain relationship with its cross-section microstructure. Though the left and right shells have the same microstructure, they showed different antiwear performance, indicating that the antiwear ability of the molluscan shell is also related to the surface topography. Therefore, the excellent antiwear property of the* Scapharca subcrenata* shell resides in the coupling of surface topography, microstructure, and component material.

**Figure 13:**
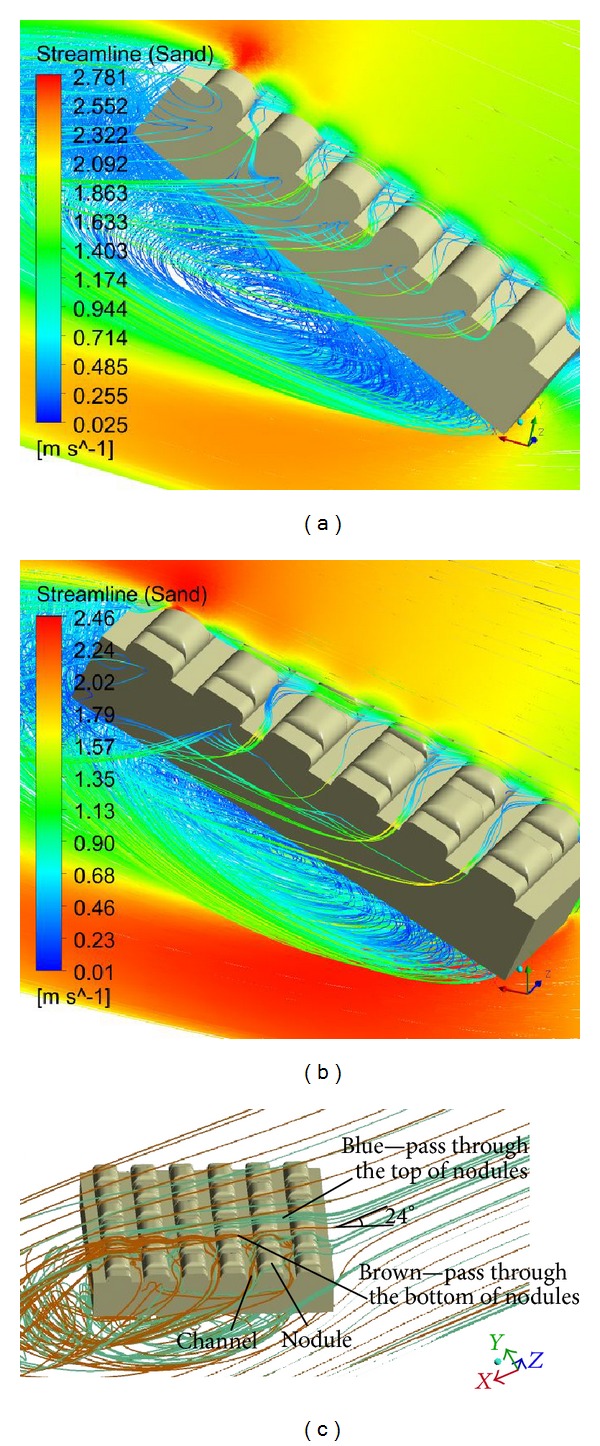
The abrasive trajectories for the two models: (a) the abrasive trajectories for the RMM; (b) the CSM; and (c) a detailed depiction of the abrasive trajectories for the CSM.

The wear resistance mechanisms of molluscan shell surface topography lay in its rib morphology and coupled structure, which comprehensively controlled the trajectories and motion methods of the abrasive elements. The trajectories of the abrasive elements are shown in Figure 13. Each rib effectively broke the continuous sliding state of the abrasives through the application of different pressures between the windward and leeward sides, generating vortices in the channels between the ribs that changed the motion of the abrasives from sliding to rolling (Figures 13(a)-13(b)). The abrasive elements that passed through the model were divided into two directions: downstream and perpendicular. In the downstream flow directional condition, vortices formed between the ribs like “rolling bearings” and triggered “sand particle cushions,” which changed the contact form and motion state between the abrasives and the model surface. This created a “scrolling” effect that significantly reduced the frictional resistance, markedly improving the wear-resisting performance. In the perpendicular to the flow directional condition, the ribs acted as guiding devices directing the abrasive scrolling while the wall shell stress was very low (Figure 10). Combining the “scrolling” and “direct” effects imbued the two models with good antiwear capabilities.

Although both models have good wear resistance characteristics, the degree of their antiwear capabilities differs. The above results suggest that the antiwear capabilities of the CSM were better than those of the RMM because the nodules on the ribs of the former changed the trajectories and motion of the abrasives (Figure 13(c)). The streamlines represented by blue and brown in Figure 12(c) refer to the abrasive trajectories passing over the tops and bottoms of the nodules, respectively. The smooth tops of the nodules prompted the abrasives to slide down and fall into the bottom of the nodules. They intervened in the abrasive flow passing through it (brown), reducing the shear stress (Table 6). However, this interference phenomenon was slightly hampered by the intensity of the vortices, which was obviously inadequate in the CSM (Figure 13(b)), so the “scrolling” effect between the ribs is decreased and the wall shear of the CSM between the ribs was higher than that in the RMM (Figure 10), but it did not affect the wear resistance properties of the whole model.

The experiments and simulations conducted in this study show that the surface topography of molluscan shells (*Scapharca subcrenata*) significantly influences their antiwear capabilities. The rib morphology and coupled structure redistribute the shear stress through changes in the trajectories and motion states of abrasive elements. Although both the RMM and CSM have predominant antiwear capabilities, the wear resistance characteristics of the latter are superior to those of the former. The CSM is more conductive to decreasing the impact velocity and energy of abrasive particles, which reduces the probability of microcrack generation, extension, and desquamation in the model. In a real-world environment, the friction sustained by* Scapharca subcrenata's *left shell is greater than that experienced by its right shell. Thus, the coupled structure of the left shell is the result of extensive evolution.

## Figures and Tables

**Table 1:** Weight loss ratio of samples varied in rib morphology and coupled structure.

Samples	Weight loss ratio (%)		*F* = 38.749*
	Rib morphology	Coupled structure	*F*_0.01_ = 13.74
Number 1	2.16	1.57	
Number 2	2.37	1.57	
Number 3	2.56	1.62	
Number 4	2.11	1.72	

Sum *T*_*i*_	9.20	6.48	*T* = 15.68
Mean ${\bar{x}}_{i}$	2.30	1.62	$\bar{x}=1.86$

*Represents a significant difference.

**Table 2:** The materials of fluid flow and the corresponding physical property parameters.

Material	Density/kg/m^3^	Particle size/mm	Dynamic viscosity/Pa·s
Quartz sand	2.32 × 10^3^	0.4	3.21
Water	1.0 × 10^3^	—	1.003

**Table 3:** The materials of the solid domain and the corresponding physical property parameters.

Material	Density/kg/m^3^	Elasticity modulus /GPa	Poisson's ratio
SiC ceramics	3.1 × 10^3^	450	0.14

**Table 4:** The surface resistances of each model on the original coordinate system.

Model	*Fx*	*Fy*	*Fz*
RMM			
Pressure force	0.0525662	0.0237315	−0.0546637
Viscous force	0.00153625	0.000265668	−0.000879406
Total force	**0.0541025**	**0.0239972**	**−0.0555431**
CSM			
Pressure force	0.0539078	0.0102197	−0.0513045
Viscous force	0.00176366	0.000446653	−0.000786766
Total force	**0.0556715**	**0.0106664**	**−0.0520913**

**Table 5:** The cutting and impact forces of each model induced by the pressure force.

Model	*F*_cutting force_	*F*_impact force_	*F*_total force_
RMM	0.079463325	0.043060403	0.090380409
CSM	0.074054938	0.031262437	0.080383293

**Table 6:** Peak stress values of the two models (Pa).

Models	Maximum tensile stress	Maximum shear stress	The von Mises stress
RMM	11476	10513	19546
CSM	11341	9614	17489

## References

[1] Barthelat F (2012). Nacre from mollusk shells: a model for high-performance structural materials. *Bioinspiration and Biomimetics*.

[2] Chen P-Y, McKittrick J, Meyers MA (2012). Biological materials: functional adaptations and bioinspired designs. *Progress in Materials Science*.

[3] Kamat S, Su X, Ballarini R, Heuer AH (2000). Structural basis for the fracture toughness of the shell of the conch Strombus gigas. *Nature*.

[4] Vincent JFV, Bogatyreva OA, Bogatyrev NR, Bowyer A, Pahl A-K (2006). Biomimetics: its practice and theory. *Journal of the Royal Society Interface*.

[5] Dai Z, Tong J, Ren L (2006). Researches and developments of biomimetics in tribology. *Chinese Science Bulletin*.

[6] Tian X, Han Z, Li X, Pu Z, Ren L (2010). Biological coupling anti-wear properties of three typical molluscan shells-*Scapharca subcrenata*, *Rapana venosa* and *Acanthochiton rubrolineatus*. *Science China Technological Sciences*.

[7] Hirvonen JP, Lappalainen R, Koskinen J, Likonen J, Pekkarinen M (1994). Wear and friction of unio crassus shell in dry sliding contact with steel. *Materials Research Society Symposia Proceedings*.

[8] Lin A, Meyers MA (2005). Growth and structure in abalone shell. *Materials Science and Engineering A*.

[9] Liang Y, Zhao J, Wang L, Li F-M (2008). The relationship between mechanical properties and crossed-lamellar structure of mollusk shells. *Materials Science and Engineering A*.

[10] Neves NM, Mano JF (2005). Structure/mechanical behavior relationships in crossed-lamellar sea shells. *Materials Science and Engineering C*.

[11] Yang W, Zhang GP, Zhu XF, Li XW, Meyers MA (2011). Structure and mechanical properties of Saxidomus purpuratus biological shells. *Journal of the Mechanical Behavior of Biomedical Materials*.

[12] Yang W, Kashani N, Li X-W, Zhang G-P, Meyers MA (2011). Structural characterization and mechanical behavior of a bivalve shell (Saxidomus purpuratus). *Materials Science and Engineering C*.

[13] Yang W, Zhang G, Liu H, Li X (2011). Microstructural characterization and hardness behavior of a biological Saxidomus purpuratus shell. *Journal of Materials Science and Technology*.

[14] Chen B, Sun ST, Peng XH, Fan JH (2009). Hierarchical microstructural characteristics and toughness mechanism of corkscrew cross infostructure of Mactra sulcataria shell. *Rare Metal Materials and Engineering*.

[15] Heinemann F, Launspach M, Gries K, Fritz M (2011). Gastropod nacre: structure, properties and growth—Biological, chemical and physical basics. *Biophysical Chemistry*.

[16] Sun JM, Guo WL (2009). Chemical-mechanical stability of the hierarchical structure of shell nacre. *Science in China Series G*.

[17] Tong J, Wang H, Ma Y, Ren L (2005). Two-body abrasive wear of the outside shell surfaces of mollusc *Lamprotula fibrosa* Heude, *Rapana venosa* Valenciennes and Dosinia anus Philippi. *Tribology Letters*.

[18] Jia X, Ling X, Tang D (2006). Microstructures and friction-wear characteristics of bivalve shells. *Tribology International*.

[19] de Paula SM, Silveira M (2009). Studies on molluscan shells: contributions from microscopic and analytical methods. *Micron*.

[20] Stempflé P, Pantalé O, Rousseau M (2010). Mechanical properties of the elemental nanocomponents of nacre structure. *Materials Science and Engineering C*.

[21] Yan Z, Chai X, Shen S, Chen D, Wang Z, Ma Y (2012). Tribological properties of Cyclina sinensis shell cuticle. *Transactions of the Chinese Society of Agricultural Machinery*.

[22] Zhang R, Lu ZZ, Li JJ (2010). Abrasive wear of geometrical surface structures of *Scapharca subcrenata* and burnt-end ark against Soil. *Advantage in Natural Science*.

